# Regulation of Chk1

**DOI:** 10.1186/1747-1028-4-8

**Published:** 2009-04-29

**Authors:** Claudia Tapia-Alveal, Teresa M Calonge, Matthew J O'Connell

**Affiliations:** 1 Department of Oncological Sciences, Mount Sinai School of Medicine, New York, NY 10029, USA; 2 Present Address: Centro de Investigación del Cáncer, Campus Miguel de Unamuno 37007 Salamanca, Spain

## Abstract

Chk1 is a serine/threonine protein kinase that is the effector of the G2 DNA damage checkpoint. Chk1 homologs have a highly conserved N-terminal kinase domain, and a less conserved C-terminal regulatory domain of ~200 residues. In response to a variety of genomic lesions, a number of proteins collaborate to activate Chk1, which in turn ensures that the mitotic cyclin-dependent kinase Cdc2 remains in an inactive state until DNA repair is completed. Chk1 activation requires the phosphorylation of residues in the C-terminal domain, and this is catalyzed by the ATR protein kinase. How phosphorylation of the C-terminal regulatory domain activates the N-terminal kinase domain has not been elucidated, though some studies have suggested that this phosphorylation relieves an inhibitory intramolecular interaction between the N- and C-termini. However, recent studies in the fission yeast *Schizosaccharomyces pombe *have revealed that there is more to Chk1 regulation than this auto-inhibition model, and we review these findings and their implication to the biology of this genome integrity determinant.

## Review

### A little history: control of entry into mitosis and the identification of chk1

The cell cycle is an orderly progression driven by the activities of the cyclin-dependent kinases (CDK) that control the transitions from G1 in S-phase, and from G2 into mitosis. The G2/M transition is particularly ancient in origin and is controlled by a universal mechanism common to virtually all eukaryotes [1]. Cdc2 (also known as Cdk1) is the mitotic CDK, and its activity is reliant upon binding to the cyclically expressed A- and B-type cyclins. To ensure that the transition from G2 into mitosis is a rapid switch, Cdc2 molecules that bind to cyclin partners are rapidly inactivated by inhibitory tyrosine phosphorylation on residue 15 (Y15). This inhibitory phosphorylation is catalyzed by the Wee1-family of kinases. During G2, Y15 phosphorylated Cdc2-Cyclin complexes accumulate, and are maintained in this inactive state until conditions appropriate for mitotic entry are completed. Given the highly mechanical and irreversible nature of mitosis, achieving appropriate cell mass, the completion of DNA replication and the absence of genomic lesions are crucial criteria that must be met for the cell to commit to mitotic entry. Once these conditions are met, Cdc2 is rapidly activated by dephosphorylation of Y15, catalyzed by the Cdc25-family of phosphatases [2]. This becomes a "point of no return", and the cells are then committed to pass through mitosis, where proteolysis of the cyclins resets the system for the subsequent cell cycle [3].

To ensure cells do not enter mitosis prematurely, checkpoints overlay the core cell cycle machinery to ultimately control Cdc2 activation. DNA damage in S-phase or G2 delays mitotic entry, and the pioneering work of Weinert and Hartwell in *Saccharomyces cerevisiae *discovered a number of radiation sensitive mutants (the *RAD *genes) that were defective in mounting a checkpoint-mediated delay to mitosis [4]. Work in *S. pombe *by a number of laboratories, most notably by Tony Carr and colleagues, also identified checkpoint genes among radiation sensitive mutants [5-9]. One of these, *rad27 *[6], was the same gene as *chk1*, a gene that was previously identified by Nancy Walworth, and shown to encode a serine/threonine protein kinase required for DNA damage checkpoint arrest [8].

**Figure 1:**
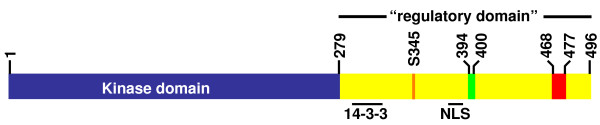
***S. pombe *Chk1 domain structure**. Chk1 has an N-terminal kinase domain (blue) and a C-terminal regulatory domain (yellow). Regions required for 14-3-3 interaction and nuclear localization [42] are indicated below the schematic. The position of S345 (orange), the site of activating phosphorylation is shown, as are the two regions of the C-terminal domain conserved across species that have been analyzed by mutagenesis [45]. These are "region 1" (green; residues 394–400, RLTRFYS in *S. pombe*, RMTRFYS in humans), and "region 2" (red; residues 468–477, GDPLEWRKFF in *S. pombe*, GDPLEWRKFY in humans).

Homologs of this kinase have subsequently been identified in all eukaryotes. They share a highly conserved N-terminal kinase domain, and a C-terminal domain that while not well conserved, is ~200 residues in all species, contains two regions of conserved sequences, and has therefore been described as a regulatory domain (Figure 1). How Chk1 is regulated has turned out to be a remarkably complex series of events, involving many upstream elements, though most of these have now been identified. What is still lacking is an understanding of the precise molecular mechanisms of Chk1 activation. Here, we review recent progress focusing on Chk1 function and regulation in *S. pombe*, which has continued to provide an excellent model for studying this pathway and cell cycle regulation relevant to all eukaryotes.

### Signaling upstream and downstream of Chk1

In the initial study of Chk1, Walworth showed that its overexpression alone caused a G2 cell cycle arrest, and this did not require any of the other checkpoint *rad *genes, suggesting it was a downstream element in a putative signaling cascade [8]. As Y15 phosphorylation of Cdc2 is so critical to control mitotic entry, it was perhaps not surprising that this was the mechanism by which Chk1 delays cells in G2 [10,11]. To achieve this, Chk1 phosphorylates Wee1, stabilizing the protein to increase cellular pools of Wee1 [12], and also Cdc25, where phosphorylation is inhibitory over catalytic activity and also affects sub-cellular localization [13,14]. Cells that lack both Wee1 and Cdc25 are viable, and still utilize the regulated abundance of cyclins to control cell cycle progression. Such cells, however, lack a checkpoint response to DNA damage, and also fail to respond to Chk1 overexpression [12], suggesting these molecules are the key substrates for Chk1, at least in *S. pombe*.

The first clue to Chk1 activation came from the observation that DNA damage led to a phosphorylation of Chk1 that was dependent on all the upstream checkpoint genes [15]. From several studies in diverse experimental systems, the events leading to Chk1 phosphorylation have been delineated, and extensively reviewed [16-19]. Briefly, primary lesions are converted into single stranded DNA (ssDNA), which is rapidly coated by the ssDNA-binding protein Replication Protein A (RPA). This acts as a landing pad for Rad26 (ATRIP in humans) [20], together with its associated kinase, Rad3 (ATR in humans). Independently, the PCNA-related 9-1-1 complex, comprised of Rad9, Rad1 and Hus1, is loaded onto these sites by a variant of Replication Factor C (RFC), in which the large sub-unit is replaced by Rad17 [21]. Two BRCT-domain mediator proteins, Cut5 and Crb2 (TopBP1 ad 53BP1 in humans), are also recruited to the complexes, and finally Chk1 is recruited into the checkpoint complex via an interaction with Crb2 [22]. This brings Chk1 into proximity to Rad3, which then phosphorylates Chk1 in Serine 345 (S345) in the C-terminal regulatory domain [23,24]. Human Chk1 contains an additional phosphorylation site at S317 [25,26], but the analogous site in *S. pombe *Chk1 (T323) is not required for Chk1 activation [24].

### Activation and inactivation of Chk1

Phosphorylation of S345 activates Chk1 kinase activity ~5–10-fold over a basal activity that is clearly measurable [27-29]. Whether this basal activity plays a significant role in regulating cell cycle progression has not been rigorously tested, but a S345A mutant grossly resembles a null allele of *chk1 *(*chk1*Δ) suggesting any effect is minor compared to the induced activity [24]. Curiously, acidic substitutions at S345 are not phospho-mimetic, and indeed inactivate Chk1 and phenocopy S345A (and *chk1*Δ) [24].

Chk1 activation is extremely rapid, and the magnitude of activation is saturated at relatively low levels of DNA damage. However, the duration of the checkpoint arrest is clearly dose-dependent, suggesting that Chk1 exists in either an "on or off" state [27]. The precise signal to inactivate Chk1 is not known, but thought to derive from completion of DNA repair. Inactivation of Chk1 during a DNA damage induced checkpoint arrest is sufficient to send cells immediately into mitosis [27]. In *S. pombe*, one of two type 1 Protein Serine/Threonine Phosphatases (PP1), Dis2, is both necessary and sufficient to dephosphorylate S345 and inactivate Chk1 [28,30]. While PP1 phosphatases have also been shown to inactivate Chk1 in human cells [31], other phosphatases and even other mechanisms have been proposed [31-33], and as seen in *S. pombe*, up-regulating S345 dephosphorylation also abrogates a checkpoint arrest.

Remarkably, although Chk1 is and must be inactivated for cells to recover from the checkpoint arrest, Chk1 is reactivated in the following cell cycle without apparent DNA damage, and yet the cells cycle normally [27,28]. This apparent paradox may be explained if DNA damage inactivates a Chk1 antagonist, but in the following cell cycle such an antagonist is active and negates the activated Chk1. Candidate antagonists in this scenario are the Cdr kinases Cdr1 and Cdr2. Like Chk1, Cdr kinases phosphorylate Wee1, but unlike the activation of Wee1 by Chk1, Cdr kinases are Wee1 inhibitors [34-38]. The inactivation of both of these kinases renders cells hypersensitive to Chk1 overexpression, and conversely the overexpression of Cdr1 renders cells checkpoint defective and insensitive to Chk1 signaling [39]. Whether Cdr kinase homologs are Chk1 antagonists in other systems, and whether they are themselves regulated by DNA damage remains to be determined.

### An auto-inhibitory model to control Chk1 function

**Figure 2:**
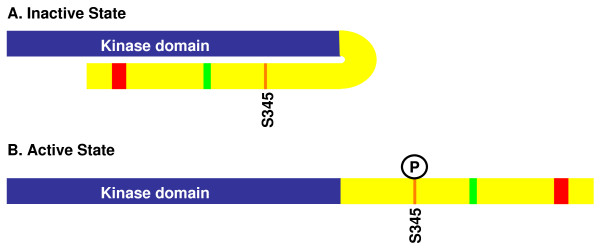
**Auto-inhibition model of Chk1 regulation**. A. Using the same color scheme as in Figure 1, in its inactive (unphosphorylated) state, the C-terminal domain physically interacts with the kinase domain, precluding it from phosphorylating substrates. B. This intramolecular interaction is blocked by S345 phosphorylation, opening the molecule to uncover the kinase domain.

Clearly S345 must be phosphorylated for Chk1 to be active, but how does this modification to the C-terminal domain activate the N-terminal kinase domain? The structure of the catalytic domain (but not the full-length protein) of human Chk1 has been solved, and it is in an open conformation; that is, it does not require modification to adopt the fold of an active kinase [40]. Moreover, the kinase activity of the isolated catalytic domain is substantially elevated over the full-length molecule, which suggested that the C-terminal domain might be auto-inhibitory through an interaction with the catalytic domain, and perhaps such an interaction is disrupted by phosphorylation (Figure 2).

This model has been tested using ectopically expressed fragments of Chk1 in Xenopus oocyte extracts, and indeed this study showed that separately expressed N- and C-terminal domains physically interact [41]. Consistent with the model of activation by phosphorylation, phospho-mimetic substitutions at the ATR phosphorylation sites, or treatment with aphidicolin (a DNA polymerase alpha inhibitor that activates Chk1), disrupted the interaction between separately expressed N- and C-terminal domains. However, in the context of full-length Chk1, acidic substitutions did not activate Chk1's kinase activity, and expression of the C-terminal domain did not activate or interact with the full-length protein [41]. Further, unpublished work from our laboratory using similar experiments overexpressing fragments of Chk1 in *S. pombe *have failed to show N- and C-terminal domain interactions, and we have observed no effect of overexpression of the C-terminal domain on the function of the endogenous full-length protein. Therefore, while auto-inhibition by N- and C-terminal domain interaction may be a component of Chk1 regulation, it appears the mechanism of activation may be more complex.

### Chk1 has positive regulatory elements in the C-terminal domain

From a series of studies over the last 16 years from several groups, 45 independent mutant alleles of *S. pombe chk1 *have been characterized (Table 1) [6,8,9,24,27,42-46]. These have come from classical screens, site-directed mutagenesis and simple truncations. For the later, given the data with human Chk1 *in vitro *[40], and with *Xenopus *Chk1 in oocyte extracts [41], a prediction would be that C-terminal truncations should activate Chk1 and cause a G2 cell cycle arrest. However, a series of truncations constructed by our laboratory and spanning the entire C-terminal domain to just the final 11 residues completely inactivate the protein in terms of its ability to rescue the functional defects of a *chk1*Δ allele [45]. The C-terminal domain includes a nuclear localization sequence and a 14-3-3-interaction domain [42], but truncations that leave these intact are also non-functional. Therefore, the C-terminal domain must contain additional sequences necessary for function *in vivo*.

**Table 1:** Mutant alleles of *S. pombe chk1*

Allele	Mutation	Reference
*chk1*Δ	Null	[6,8]

*chk1-T15*	ND^1^	[6]

*chk1-1*	E92D	[46]

*chk1–2*	I484T	[46]

*chk1-NC18*	ND	[9]

*chk1-NC73*	ND	[9]

*chk1-NE3*	ND	[9]

-	K38A	[44]

-	G108S	[44]

-	F118L, S311F	[44]

-	N142D	[44]

-	L144P	[44]

-	D155G	[44]

-	D155A	[44]

-	D155E^2^	[44]

-	D363G, D469G	[44]

-	S345A	[23,24]

-	S345D	[24]

*chk1-ts1*	E472D^3,7^	[27]

*chk1-ts2*	E111R^4^	[27]

*chk1-ts3*	L151S^4^	[27]

*chk1-ts4*	I389S^4^	[27]

*chk1-ts5*	ΔI484^4^	[27]

-	L291A^5^	[42]

-	L295A^5^	[42]

-	L299A^5^	[42]

-	L303A^5^	[42]

*chk1–4A*	K377A, R378A, K381A, K382A^6^	[42]

-	D363G	[43]

-	D469G	[43]

-	Δ280–496	[45]

-	Δ327–496	[45]

-	Δ393–496	[45]

-	Δ444–496	[45]

-	Δ467–496	[45]

-	Δ486–496	[45]

-	Δ359–361^3,7^	[45]

-	R394A^7^	[45]

-	R394A, R397A	[45]

-	F398A, Y399A	[45]

-	Δ394–400	[45]

-	E472A	[45]

-	ΔE472	[45]

-	ΔF477	[45]

-	Δ473–477	[45]

-	G468A, D469A, P470A, E472A	[45]

Notes: ^1^Not determined; ^2^dominant-negative when overexpressed; ^3^temperature sensitive loss-of-function; ^4^temperature sensitive loss-of-function when expressed from a multi-copy plasmid, but non-functional when expressed from the *chk1 *locus; ^5^disrupts 14-3-3 binding; ^6^blocks nuclear localization; ^7^gain-of-function when expressed from the attenuated *nmt1 *promoter.

A number of non-functional alleles are mutations in conserved residues within the kinase domain, and can be easily explained by a reduction or lack of kinase activity. However, there are also many non-functional mutant alleles that are single amino acid substitutions in the C-terminal domain. Some of these are in the two highly conserved regions, and most were generated by site-specific mutagenesis based on homology between human and *S. pombe *Chk1. However, several loss-of-function alleles of *S. pombe chk1 *are in non-conserved regions of the C-terminal domain, suggesting that they alter structure to a point that disrupts function. Unfortunately, no structural data for the C-terminal domain has been reported on which these mutants can be mapped. Nevertheless, these non-functional C-terminal point mutants support the interpretation of the truncation mutations; that is, regardless of whether it is inhibitory or not, the C-terminal domain is critical for Chk1 function *in vivo*.

### Activating mutations in the C-terminal domain

Our laboratory has also isolated activating mutations in the conserved regions of the C-terminal domain [45]. The analysis of these alleles supports the auto-inhibitory model, functionally separating them from the inactivating mutations within the very same domains. Modest overexpression of Chk1 from a heterologous attenuated *nmt1 *promoter has no affect on cell cycle progression. However, the expression of substitutions at two conserved residues, R394A and E472D, from the same attenuated *nmt1 *promoter causes a lethal G2 cell cycle arrest. Each of the activating mutations in the C-terminal domain required kinase activity to elicit a cell cycle arrest, but all were independent of S345 phosphorylation. The simplest interpretation is that these activating C-terminal mutations are having the same net biochemical effect on Chk1 as does S345 phosphorylation.

Remarkably, the E472D mutation corresponded to a temperature-sensitive loss-of-function allele (*chk1-ts1*) we had previously isolated [27]. When expressed from its own promoter, Chk1-E472D is a less stable protein, suggesting that while this mutation imparts an activated conformation, this is also destabilizing and the protein is more rapidly degraded.

Several second site mutations within Chk1 that suppress the gain-of-function conferred by the E472D mutation, but result in a fully functional protein were selected [45]. One of these suppressor mutations, an in frame deletion of residues 359–361 (Δ359–61), is itself a gain-of-function mutant when mildly overexpressed, and like E472D, confers a temperature sensitive loss-of-function when expressed from the endogenous locus. Thus, Δ359–61 and E472D show mutual suppression, where the combination of two activating mutations in the same protein likely confers a more wildtype conformation.

**Figure 3:**
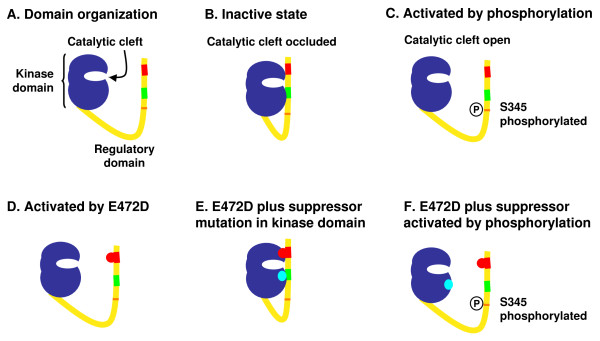
**Model of regulatory effect of Chk1 mutations**. Using the same color scheme as in Figure 1: A. a postulated domain organization is shown. B. In its inactive state, the C-terminal domain interacts with the kinase domain to occlude the active site catalytic cleft. C. Phosphorylation opens the molecule to expose the catalytic cleft. D. Activating mutations such as E472D (red, in region 2) block the intramolecular interactions that occlude the catalytic cleft, activating Chk1's kinase activity. E. The E472D suppressor mutations in the kinase domain (Cyan) restore the intramolecular interaction to again occlude the active site. F. The molecule containing both E472D and a suppressor mutation can still be opened by S345 phosphorylation, restoring normal regulation in response to DNA damage.

Several of the other E472D suppressors clustered to regions in the catalytic domain. When the positions of these residues are mapped onto the crystal structure of the human enzyme, each cluster mapped onto loops on either side of the catalytic cleft on the face of the protein that interacts with substrate. We speculate that, when Chk1 is in an inactive conformation, these regions within the catalytic domain interact with the C-terminal domain, and that this interaction occludes the active site. The E472D mutation may destabilize this interaction, but the suppressor mutants restore the interaction to return the molecule to a wildtype conformation (Figure 3).

Further physical analyses will be required to test this model. The end result is that this model is essentially a slightly refined version of the auto-inhibition model proposed by Sagata and colleagues, and as such, also does not take into account why the C-terminal domain is essential for function *in vivo*. However, the collection of mutants of defined function might enable us to separate the negative and positive regulatory elements within this domain, and move towards a detailed view of the mechanism of activation (and inactivation) for Chk1.

## Conclusion

Chk1 was first identified 16 years ago, and in the interim we have learned much about the events leading to its activating phosphorylation. At a mechanistic level, we have learned comparatively little regarding how this phosphorylation actually elevates Chk1 kinase activity. Because the G2 checkpoint appears to be required for tumor cell viability, there has been significant interest in inhibiting Chk1 as a means to treat cancer, though most of these studies have relied on ATP analogs [47]. Determining more precise mechanisms of Chk1 activation is thus not only important for the biology of this highly conserved pathway, but may also have profound implications in the design of Chk1-based anti-cancer therapeutics.

## Competing interests

The authors declare that they have no competing interests.

## Authors' contributions

CTA, TMC and MOC drafted the manuscript. All authors read and approved the final manuscript.
